# Multifunctional Roles of Plant Cuticle During Plant-Pathogen Interactions

**DOI:** 10.3389/fpls.2018.01088

**Published:** 2018-07-25

**Authors:** Carmit Ziv, Zhenzhen Zhao, Yu G. Gao, Ye Xia

**Affiliations:** ^1^Department of Postharvest Science of Fresh Produce, Agricultural Research Organization – the Volcani Center, Rishon LeZion, Israel; ^2^Department of Plant Pathology, The Ohio State University, Columbus, OH, United States; ^3^The Ohio State University South Centers, Piketon, OH, United States; ^4^Department of Horticulture and Crop Science, The Ohio State University, Columbus, OH, United States

**Keywords:** plant cuticle, cutin and wax, plant-pathogen interaction, plant defense, cuticle-cell wall continuum, hormone signaling

## Abstract

In land plants the cuticle is the outermost layer interacting with the environment. This lipophilic layer comprises the polyester cutin embedded in cuticular wax; and it forms a physical barrier to protect plants from desiccation as well as from diverse biotic and abiotic stresses. However, the cuticle is not merely a passive, mechanical shield. The increasing research on plant leaves has addressed the active roles of the plant cuticle in both local and systemic resistance against a variety of plant pathogens. Moreover, the fruit cuticle also serves as an important determinant of fruit defense and quality. It shares features with those of vegetative organs, but also exhibits specific characteristics, the functions of which gain increasing attention in recent years. This review describes multiple roles of plant cuticle during plant-pathogen interactions and its responses to both leaf and fruit pathogens. These include the dynamic changes of plant cuticle during pathogen infection; the crosstalk of cuticle with plant cell wall and diverse hormone signaling pathways for plant disease resistance; and the major biochemical, molecular, and cellular mechanisms that underlie the roles of cuticle during plant-pathogen interactions. Although research developments in the field have greatly advanced our understanding of the roles of plant cuticle in plant defense, there still remain large gaps in our knowledge. Therefore, the challenges thus presented, and future directions of research also are discussed in this review.

## Introduction

Plant cuticle is the outermost layer of plants, which covers leaves, fruits, flowers, and non-woody stems of higher plants. It protects plants against drought, extreme temperatures, UV radiation, chemical attack, mechanical injuries, and pathogen/pest infection. It also provides mechanical support and serves as a barrier against organ fusion (Yeats and Rose, 2013; Kim et al., 2017).

**FIGURE 1:**
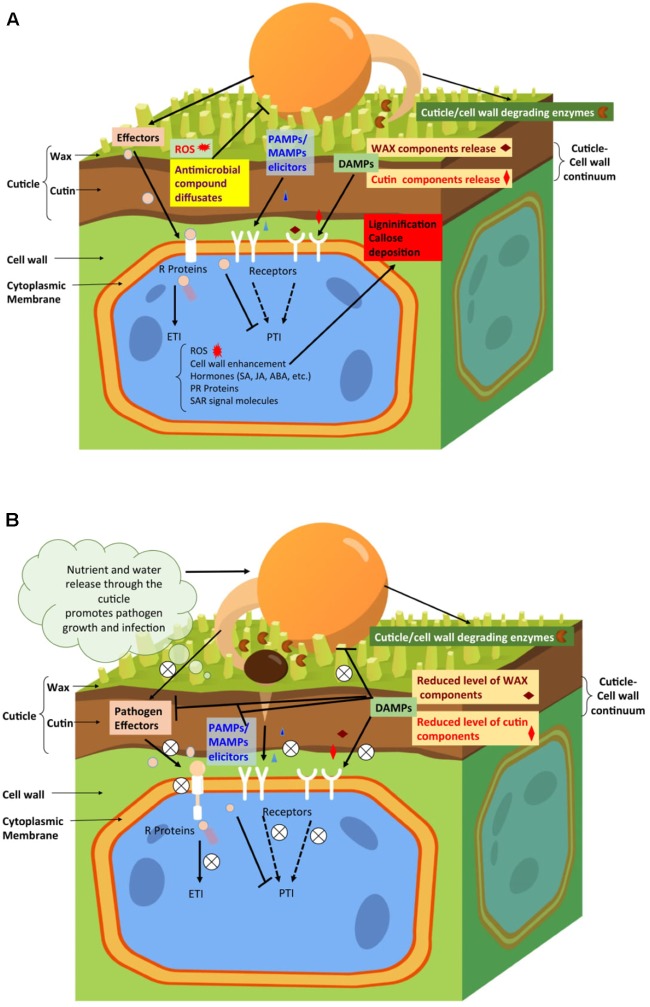
Hypothetical model representing the involvement of plant cuticle during plant interactions with various pathogens. **(A)**. During pathogen infection of resistant plants, the specific cuticle properties may affect the outcome of the interaction. For example, in response to infection, plants with a more permeable cuticle could release defense signals much faster. These signals comprise certain cutin monomers or wax components (e.g., DAMPs), elicited by cuticle- or/and cell wall-degrading enzymes secreted by the pathogens. Plants with a more permeable cuticle could also respond faster to the pathogen elicitors (e.g., MAMPs/PAMPs) and effectors in activating the plant disease resistance through PTI and ETI, respectively. Both PTI and ETI activate plant disease resistance, but ETI is a stronger and more efficient response through the interactions of R proteins with pathogen effectors. The ETI response might include cuticle and cell wall remodeling, release of antimicrobial compounds and ROS, and production of defense hormones, PR proteins, and SAR signaling molecules. **(B)**. During pathogen infection on susceptible plant hosts, the enhancement of specific cuticle properties, such as permeability, might lead to reduced levels of certain cutin monomers and/or wax components that could repress the virulence and pathogenicity of pathogens and their effectors. These specific cutin monomers and/or wax components could serve as signals to activate plant defense locally and/or systemically. The enhanced cuticle permeability might also disrupt the elicitors (MAMPs/PAMPs) that could be detected by plant receptors to trigger the defense. The increased release of water and dissolved nutrient compounds at the leaf surface could enhance pathogen fitness and support its growth and pathogenicity, and facilitate pathogens’ entry through the plant cuticle and/or cell wall and the injection of their virulent and pathogenic effectors. All these effects will impair the ability of plants to activate their defense mechanisms and eventually result in their susceptibility to the pathogens.

Plant cuticle mainly comprises a matrix of cutin (an insoluble polyester) and embedded wax (soluble lipids) (**Figure 1**) (Kunst and Samuels, 2009). The wax and cutin compositions of plant cuticle can vary widely among plant species and various organs (Yeats and Rose, 2013). The cuticle of each organ has specific characteristics, for example, fruit cuticle is generally thicker than leaf cuticle and lacks stomata. Because fruit cuticle is a critical modulator of postharvest fruit quality, such as its effects on fruit water retention (Kosma et al., 2010), responses to physical and biological stresses (Kosma et al., 2009), and firmness (Matas et al., 2009; Lara et al., 2014; Vallarino et al., 2017), it is attracting increasing research attention.

For most plant species, cutin polymers mainly contain linked C16 and C18 esterified and oxygenated fatty acids (FAs), small amounts of glycerol, phenyl-propanoids, etc., (Heredia, 2003). The cuticular waxes are complex mixtures, which mainly consist of various primary and secondary alkanes, alcohols, aldehydes, ketones, and esters derived from very-long-chain FAs (C20–C34) (Raffaele et al., 2009; Malinovsky et al., 2014). Thus, FAs are the main precursors for biosynthesis of both cutin and wax, which occur mainly in plant chloroplasts. The resulting FAs are exported to endoplasmic reticulum (ER), across plasma membrane and cell wall of epidermal cells, and are deposited at the nascent cuticular membrane, where they form cutins, waxes, and suberins (complex polyesters) (Kunst and Samuels, 2009; Yeats and Rose, 2013).

There has been significant progress in the past 20 years on identification and characterization of the genes involved in plant cutin and wax biosynthesis (Suh et al., 2005; Lee and Suh, 2013; Fich et al., 2016). Also, factors involved in transportation of precursors of cutins and waxes, such as acyl-CoA-binding proteins (ACBPs) (Xia et al., 2012; Xue et al., 2014), lipid transfer protein (LTP) (Deeken et al., 2016), and ABC transporter (Luo et al., 2007), were characterized. Furthermore, several transcription factors, such as AP2, MYB94, MYB96, MYB16, and zinc-finger NFXL2 were found to play critical roles in regulating biosynthesis of plant cutin and wax (Fich et al., 2016; Lee et al., 2016). There is increasing evidence that cuticle is not merely a physical layer that protects plants; it appears to be actively involved in plant defense and signaling pathways for growth and development (Raffaele et al., 2009; Javelle et al., 2011; Aragón et al., 2017). Various studies showed that plant cuticle could function in the first layer of plant defense pattern-triggered immunity [PTI, including MAMP (microbe-), PAMP (pathogen-), and DAMP (damage-) associated molecular patterns] and the second, stronger layer of plant defense effector-triggered immunity (ETI). Thus, it serves to activate local and systemic acquired resistance against diverse pathogens (**Figure 1**) (Heredia, 2003; Xia et al., 2009; Aragón et al., 2017). The plant genes and transcription factors involved in cuticle biosynthesis/signaling and associated plant-microbe interactions were well summarized in several informative reviews (Chassot and Métraux, 2005; Muller and Riederer, 2005; Reina-Pinto and Yephremov, 2009; Lee and Suh, 2013; Serrano et al., 2014; Fich et al., 2016; Aragón et al., 2017). Studies of the plant microbial community associated with cuticular surface were well reviewed by Aragón et al. (2017). These studies, although relevant, will not be further discussed in this mini-review because of space limitation.

This review briefly summarizes the multifunctional roles of plant cuticle during plant-pathogen interactions, with emphasis on: dynamic changes of plant cuticle and their regulation; the crosstalk of cuticle with cell wall and diverse hormone signaling pathways for plant defense; and major biochemical, molecular, and cellular mechanisms that underlie the roles of cuticle during plant-pathogen interactions. The present review also discusses the challenges and future directions for related study, such as novel genes and mechanisms involved in transportation, regulation, assembly, and deposition of cuticle precursors/signals, and the associated plant-pathogen interactions.

## Plant Cuticle and Cell Wall Form a Continuum at the Plant Surface, Which is Dynamic and Responsive to Diverse Pathogen Infections

Plant cell wall spans between cuticle and epidermis’ cell membrane and forms a continuum with cuticle (**Figure 1**; Nawrath et al., 2013). The main components of plant cell wall are cellulose, hemicellulose, pectin, and lignin. They are involved in maintaining cell shape, supporting plant growth and development, and protecting plants from biotic and abiotic stresses (Keegstra, 2010). These cell wall polysaccharides can be incorporated into cutin matrix and thereby determine the elasticity and stiffness of the whole cuticle (Lopez-Casado et al., 2007). Cuticular wax forms a barrier to transpiration (Schonherr, 1976) and cutin matrix contributes to its mechanical strength (Kolattukudy, 1980).

Cuticle and cell wall play overlapping roles in plants; in addition to their roles as passive physical and chemical barriers, they actively function together in regulating the movement of molecules into and out of plants. They also play critical roles in relaying signals inside and outside plant cells in response to diverse stimulations for plant growth and development, and resistance to biotic and abiotic stresses (Segado et al., 2016). Both cell wall and cuticle can expand and change their compositions during various plant growth and development stages and in response to varying environmental conditions (Bargel and Neinhuis, 2005; Underwood, 2012). During plant-pathogen interactions, plant cuticle and cell wall compositions might be affected by pathogens, and conversely, pathogens can sense plant-surface components and adjust their pathogenesis and virulence accordingly. At an early stage of infection, phytopathogenic fungi can synthesize hydrolytic enzymes, such as cutinases, esterases, and lipases, which directly target cuticle and thereby play key roles in pathogenic infection (Berto et al., 1999; Garrido et al., 2012; Leroch et al., 2013; Wang et al., 2017). For instance, the fungal pathogen *Fusarium oxysporum* secretes cutinases, which degrade plant leaf cuticle and produce basal levels of cutin monomers to facilitate pathogen adhesion to hosts at an early stage of infection. Once a pathogen senses the resulting plant cuticle monomers, it can expand its cutinase activity to facilitate further penetration and infection in the plant cuticle layer (Woloshuk and Kolattukudy, 1986). Plant leaf cutin components, such as hexadecanediol in rice, could induce the germination and appressorium differentiation of the rice blast fungus *Magnaporthe grisea* (Gilbert et al., 1996) and spore germination and cutinase expression of the gray mold fungus *Botrytis cinerea* (Leroch et al., 2013). Furthermore, plant leaf wax components, such as very-long-chain C26 aldehydes of maize (*Zea mays*) could affect spore germination and penetration of barley powdery mildew *Blumeria graminis* f.sp. *hordei* (Hansjakob et al., 2011). Interestingly, many fungi, such as *Botrytis* (Castillo et al., 2017), *Phytophthora* (Blackman et al., 2014), and *M. oryzaea* (Quoc and Chau, 2017) also secrete cell wall-degrading enzymes (CAZymes) even before they penetrate the cuticle, which is further evidence that the cuticle/cell wall continuum is a significant factor in plant-pathogen interactions.

Conversely, plants can recognize the attachment of pathogens and react very quickly to the elicitors (MAMPs/PAMPs) they produce. DAMPs, the pathogen-infection generated plant-degradation products, such as cutin monomers and cell wall oligosaccharides, also serve as signals that activate plant defenses against pathogen (Underwood, 2012; Malinovsky et al., 2014). For instance, tomato fruit cuticle was remodeled in response to infection of the fungal pathogens *Colletotrichum gloeosporioides*, and fruit cuticle biosynthesis was up-regulated during appressorium formation even before penetration (Alkan et al., 2015). Another example, during infection of citrus petals by *Colletotrichum acutatum*, the epidermal cells responded to the pathogen by increasing lipid synthesis and deposition of cuticle- and cell wall-associated compounds, and this eventually altered the cuticle structures (Marques et al., 2016). In Arabidopsis, instead of cuticle, the crown galls caused by bacterium *Agrobacterium tumefaciens* infection are covered with suberin, which needs transportation of FAs to support its synthesis (Deeken et al., 2016).

In addition to wax and cutin, plant cuticle contains terpenoids and flavonoids, which have antifungal activities (Arif et al., 2009; Zacchino et al., 2017). Biosynthesis of these phenylpropanoids and flavonoids follows the formation of cuticular lipids (Mintz-Oron et al., 2008), which could be induced in response to environmental signals, such as *C. gloeosporioides* infection, thereby activating plant defenses in tomato and mango fruits (Alkan et al., 2015; Sivankalyani et al., 2016).

## Cuticle Permeability, Composition, and Multiple Roles During Plant-Pathogen Interaction

Plant cuticle also plays critical roles in plant defense against diverse bacterial and fungal pathogens, most of which use natural openings, such as stomata and hydathodes in leaves, or lenticels in fruits to enter plants without directly penetrating the cuticle layer (Buxdorf et al., 2014). The integrity and permeability of cuticle are very important for its function during plant-pathogen interactions; for instance, a more permeable plant cuticle could lead to either resistance or susceptibility to pathogen infections.

Previous studies on cuticle-defective mutants of Arabidopsis and tomato, such as *CYP86A2* (cytochrome P450-dependent oxidases) (Xiao et al., 2004), *LACS2 (long-chain Acyl-CoA synthetases)* (Bessire et al., 2007; Tang et al., 2007), *PER57* (over-expressed *peroxidase 57*) (Survila et al., 2016), BODYGUARD (alpha-beta hydrolase) (Céline et al., 2007), and DEWAX transcription factor (Suh and Go, 2014; Ju et al., 2017) were shown to increase leaf cuticle permeability. Interestingly, these mutants improved plant resistance against the fungal pathogen *B. cinerea* but increased susceptibility to the bacterial pathogen *Pseudomonas syringae*. However, not all cuticle-related mutants showed resistance to *B. cinerea*: for example, our previous study found that Arabidopsis *ACP4* (acyl carrier protein 4) and *GL1* (*GLABROUS 1)* mutants had decreased levels of cutin and wax components, enhanced the permeability of leaf cuticle, and increased susceptibility to both pathogens (Xia et al., 2009, 2010). In other studies, mutations in SHINE transcription factors resulted in altered cuticle, which led to plant susceptibility to *B. cinerea* infection (Sela et al., 2013; Buxdorf et al., 2014).

Several mechanisms may account for the increased leaf resistance to *B. cinerea* and other pathogens when plant cuticle permeability increases:

(1) Release of certain cutin monomers or wax components that function as signals to activate plant disease resistance (Aragón et al., 2017);
(2) Release of antifungal diffusates and ROS (reactive oxygen species) that inhibit pathogen growth and infection at the surface (L’Haridon et al., 2011; Sela et al., 2013);
(3) Accelerated uptake of elicitors (e.g., MAMPs/PAMPs/DAMPs) activated by pathogens for PTI; and release of avirulent effectors for ETI, which could stimulate a stronger and more efficient plant-defense response (Chassot et al., 2008; Aragón et al., 2017).

A hypothetical model of the related plant disease resistance mechanism is depicted in **Figure 1A**.

Several mechanisms were suggested by which leaf cuticle permeability could raise plant susceptibility to *P. syringae* and other pathogens:

(1) Reductions in the levels of certain cutin monomers and/or wax components that help to repress the expression and function of pathogenic effectors involved in virulence and pathogenicity, such as Arabidopsis *CYP86A2*. This gene is involved in the formation of C16 and C18 hydroxy FAs, which have direct inhibitory impacts against certain pathogens, such as *P. syringae* (Xiao et al., 2017).
(2) Reductions in the levels of cutin monomers and/or wax components that may serve as signals or receptors to activate plant defense locally and/or systemically for both PTI and ETI; in our previous study on Arabidopsis *ACP4*, the mutant *ACP4* plants significantly reduced both cutin and wax biosynthesis, thereby causing defects in both local and systemic plant immunity against *P. syringae* (Xia et al., 2009, 2010).
(3) Plant defense response impairment by disruption of the plant receptors’ perception of pathogen-generated elicitors (MAMPs/PAMPs) or over-stimulated activation of certain genes by accelerated ROS production to cause plant susceptibility and death (Sela et al., 2013).
(4) Increased availability of water and dissolved nutrient compounds on leaf surface, which would enhance pathogen fitness, growth, and pathogenicity (Schreiber et al., 2005);
(5) Changes to the stomata and the cuticle-cell wall continuum that provide an easier way for pathogens to enter plants and release their virulence effectors inside.

The hypothetical model for explaining the related mechanism in plant disease susceptibility is illustrated in **Figure 1B**.

Since *B. cinerea* is a necrotrophic pathogen and *P. syringae* is a hemi-biotrophic pathogen, the cuticle may have specific modes of action to interact with pathogens having differing life styles. The specific functions of various plant leaf cuticle components may play diverse roles in plant-pathogen interactions; however, this requires further investigation.

The role of fruit cuticle in postharvest protection from pathogens has been extensively studied. Recent findings suggest that cuticle composition, rather than its mere thickness, determines fruit response to postharvest pathogens. For instance, during fruit ripening, tomato fruit susceptibility to necrotrophic fungal pathogen infection increased (Segado et al., 2016), whereas grape berries acquired resistance to biotrophic fungal pathogen powdery mildew (*Uncinula necator*) as they grew and developed (Fich et al., 2016). Thus, the multiple roles of plant cuticle during plant-pathogen interactions can be affected by cuticle thickness, permeability, or specific cuticular components in different tissues; they also vary with differing growth stages and environmental conditions.

## Hormones are Involved in Cuticle Formation and Related Signaling During Plant-Pathogen Interactions

Hormones regulate plant growth throughout the entire life cycle, controlling cell division, elongation and differentiation, tissue pattern formation and development, and responses to the biotic and abiotic stresses (Robert-Seilaniantz et al., 2007). Significant progress has been made in studying the biosynthesis and regulation of various hormones, such as salicylic acid (SA), jasmonic acid (JA), gibberellins (GA), abscisic acid (ABA), and ethylene (ET) (Kimbara et al., 2013; De Smet et al., 2015). And the critical roles of these hormones in plant-microbe interactions were well documented (Denancé et al., 2013). The study of crosstalk between plant hormones and cuticle for its biosynthesis and related functions during stress conditions, such as pathogen infection, also has been investigated, but there remain large gaps in our knowledge.

Several plant hormones were shown to influence plant cuticle formation and stress tolerance. For instance, we found that GA_4_- and GA_7_-treated Arabidopsis plants showed increased levels of cuticular wax and cutin components, which were associated with the improved plant immunity against bacterial pathogen *P. syringae* infection (Xia et al., 2010).

Jasmonic acid, an important hormone in plant defense, is derived from the 18:3 FA (linolenic acid). Methyl-JA treatment of *Vicia sativa* seedlings was found to induce production of ω-hydroxy FAs, which were involved in cutin formation (Pinot et al., 1998). Moreover, the ω-hydroxy FAs could induce plant resistance against pathogen infection by functioning as endogenous signaling molecules; for example, they could play critical roles in barley resistance against the fungal pathogen *Erysiphe graminis f.sp. hordei* (Schweizer et al., 1996).

The Arabidopsis *RST1* (*RESURRECTION1*) gene functions importantly both in biosynthesis of cutin and wax, and in plant defense. The *rst1* mutant plants showed resistance to *B. cinerea* that was associated with the up-regulated expression levels of JA and the related defense gene PDF1.2. However, the *rst1* mutant plants exhibited down-regulated levels of SA and PR proteins, such as PR-1, which were correlated with their susceptibility to the biotrophic pathogen, *Erysiphe cichoracearum* (Mang et al., 2009).

Arabidopsis transcription factor *SHN1* mutant plants exhibited defective leaf cuticle compositions and enhanced susceptibility to *B. cinerea*. The *shn1-1D* plants accumulated high levels of H_2_O_2_, and up-regulated a large set of genes associated with senescence, oxidative stress, and defense (Sela et al., 2013). For example, the ROS-associated genes *PROPEP3* (elicitor peptide 3 precursor) and *AOX1d* (alternative oxidase gene) were highly up-regulated. *PROPEP3* had been predicted to be the amplifier for ET/JA and SA pathways, and expression of *AOX1d* was also associated with these pathways (Lin and Wu, 2004; Huffaker and Ryan, 2007). The *B. cinerea* susceptibility of *shn1-1D* plants could be excessively accelerated the generation of ROSs, such as H_2_O_2_, leading to uncontrolled, excessive defense reactions, and consequent plant sensitivity and death (Sela et al., 2013).

In addition, exogenous application of ABA can specifically stimulate the formation of cuticular components in Arabidopsis, *Lepidium sativum*, and tomato plants, and this helped to decrease plant water loss during drought (Kosma et al., 2009; Macková et al., 2013; Cui et al., 2016; Martin et al., 2017). Furthermore, a tomato ABA-deficient *sitiens (sit)* mutant with reduced ABA levels and increased cuticle permeability exhibited increased resistance against *B. cinerea*. The related disease resistance was associated not only with changes in cuticle permeability, but also with changes in cell wall compositions. For example, after pathogen infection levels of pectin methyl-esterification and various oligosaccharides were higher in mutant than in wild-type plants (Curvers et al., 2010).

The mechanisms of crosstalk between cuticle and plant hormone pathways during pathogen interactions with fruits have started to be elucidated only recently. Indeed both ABA and ET signaling play important roles in regulation of fruit cuticle biosynthesis and function (Alkan and Fortes, 2015; Leida et al., 2016; Wang et al., 2017).

The possibilities that other hormone pathways crosstalk with cuticle biosynthesis and signaling pathways are largely unknown. The interactions between plant hormones and plant cuticle in relation to response to pathogen infections need to be further investigated.

## Conclusion and Perspectives

Altogether, we have briefly summarized recent advances in our knowledge of multiple roles of plant cuticle during interactions with diverse pathogens. Research in related fields has yielded evidence that plant cuticle plays critical roles during plant-pathogen interactions. However, we are still far from fully understanding the relevant mechanisms and from developing efficient strategies to utilize the plant cuticle for plant defense. Furthermore, studies of several aspects will strengthen our understanding of the related mechanisms, which include the specific roles of the various components of cutin and wax as important factors and signaling molecules that promote either resistance or susceptibility; transmission and perception of the related factors and signals; and the crosstalk between cuticle-cell wall and hormone signaling pathways, etc. Studies of all these will provide us with more detailed knowledge to develop breeding and biotechnological approaches for enhancing cuticle function and thereby improving plant health and yield.

## Author Contributions

YX and CZ organized and wrote the whole manuscript, ZZ and YG contributed to part of the writing and improvement.

## Conflict of Interest Statement

The authors declare that the research was conducted in the absence of any commercial or financial relationships that could be construed as a potential conflict of interest.
